# Effects of a multicomponent exercise regimen on subchondral bone and cartilage in postmenopausal women with knee osteoarthritis: protocol for a randomized controlled trial

**DOI:** 10.1186/s13063-025-08928-1

**Published:** 2025-06-23

**Authors:** Ville-Markus Konola, Jari Parkkari, Juhani Multanen, Riku Nikander, Timo Rantalainen, Johanna Vesanto, Satu Pekkala, Merja Kalaja, Johanna K. Ihalainen, Benjamin Waller, Matti Munukka, Harri Sievänen, Mika Nevalainen, Hannu Kautiainen, Victor Casula, Juha Paloneva, Tommi Vasankari, Arttu Peuna, Simo Saarakkala, Miika T. Nieminen, Ari Heinonen

**Affiliations:** 1 Faculty of Sport and Health Sciences, University of Jyväskylä, PO Box 35, Viveca, Jyväskylä, 40014 Finland; 2 Faculty of Medicine and Health Technology, University of Tampere, Tampere, Finland; 3 South-Eastern Finland University of Applied Sciences, Savonlinna, Finland; 4 Wellbeing Services County of Central Finland, Jyväskylä, Finland; 5 Finnish Institute of High Performance Sport KIHU, Jyväskylä, Finland; 6 UKK Institute for Health Promotion Research, Tampere, 33500 Finland; 7 Research Unit of Health Sciences and Technology, University of Oulu, Oulu, Finland; 8 Medical Research Center Oulu, Oulu University Hospital and University of Oulu, Oulu, Finland; 9 Department of Diagnostic Radiology, Oulu University Hospital, Oulu, Finland; 10 Primary Health Care Unit, Kuopio University Hospital, Kuopio, Finland; 11 Folkhälsan Research Center, Helsinki, Finland; 12 Department of Surgery, Hospital Nova, Wellbeing Services County of Central Finland, Jyväskylä, Finland; 13 University of Eastern Finland, Kuopio, Finland; 14 Department of Diagnostic Services, Hospital Nova, Wellbeing Services County of Central Finland, Jyväskylä, Finland

**Keywords:** Osteoarthritis, Knee, Subchondral bone, Cartilage, Exercise therapy

## Abstract

**Background:**

Knee osteoarthritis (KOA) is considered a whole-joint disease that is amenable to prevention and treatment in the early stages. Exercise is among the core treatment recommendations for KOA and it has been suggested that optimal exercise regimens should improve aerobic capacity and knee extensor strength. Subchondral bone and articular cartilage are functionally paired, and information on the responses of these tissues to exercise may help in the development of efficacious and feasible exercise regimens that can potentially improve bone and cartilage properties. This article describes a clinical trial investigating the effects of a multicomponent exercise regimen on the subchondral bone and articular cartilage of the knee joint in postmenopausal women with mild KOA.

**Methods:**

A minimum of 90 postmenopausal women between the ages of 55 and 75 meeting the inclusion criteria will be recruited. After an initial assessment, the participants will be randomly assigned to two groups. The intervention group will participate in a progressive multicomponent exercise regimen, including step aerobics and resistance training alternating every 2 weeks, for 50 min three times a week for 8 months. The reference group will be conducting home exercise program representing standard rehabilitative management for KOA patients. The primary outcome measures of this trial are the 8-month changes in the biochemical composition of the knee articular cartilage measured by the T1r and T2 relaxation times from quantitative magnetic resonance imaging and subchondral bone sclerosis, density and structure as measured via cone beam computed tomography. Measurements will be performed at baseline, after the 8-month intervention period, and at 12 months of maintenance.

**Discussion:**

This RCT investigates the effectiveness of a multicomponent exercise regimen on the subchondral bone and cartilage of the knee joint and the potential interaction between these tissues. The information gained will improve our understanding of the effects of exercise on subchondral bone and the biochemical properties of articular cartilage and improve the prescription of multicomponent exercise regimens in the management of mild KOA.

**Trial registration:**

ClinicalTrials.gov NCT06173193. Retrospectively registered before completion of the recruitment on 31 October 2023, https://www.clinicaltrials.gov/study/NCT06173193.

**Supplementary Information:**

The online version contains supplementary material available at 10.1186/s13063-025-08928-1.

## Introduction

Knee osteoarthritis (KOA) is considered a whole-joint disease that is amenable to prevention and treatment in the early stages [1]. Symptoms of this disease include pain around the knee joint, loss of range of motion, grinding and popping sounds from the joint, and muscle weakness [2]. These symptoms may cause difficulties in daily activities such as walking, climbing stairs, and performing household chores, which can lead to a decreased quality of life [3]. As the prevalence of KOA is high, being approximately 23% globally in individuals aged 40 years and over [4], it places a considerable burden on individuals as well as societies [5]. Risk factors for this disease include age, obesity, female sex, repeated stress on the joint, muscle weakness, and previous knee injuries [6, 7].

In the Osteoarthritis Research Society International and European Society for Clinical and Economic Aspects of Osteoporosis, Osteoarthritis and Musculoskeletal Diseases KOA guidelines, the core set of nonpharmacological treatments includes patient education, weight loss for overweight, and a structured exercise program [8, 9]. It has been suggested that optimal exercise programs should improve aerobic capacity and knee extensor strength, and the program should be carried out three to five times a week, with each session lasting an hour [10, 11]. However, despite its broad application, the impact of exercise loading on knee joint tissues in humans is poorly understood, and the benefits of exercise for pain and physical function are small and thus of questionable clinical importance [12].

Current evidence suggests that subchondral bone is actively involved in osteoarthritis (OA) development and that bone and articular cartilage are functionally coupled either through the distribution of joint loads or the exchange of signaling molecules between the tissues [13–15]. Exercise has good potential for improving bone traits by enhancing bone mass, structure, and strength at loaded sites across the age spectrum, as well as appearing safe for articular cartilage, and thus could be an important regimen for overall joint health to reduce the progression of OA [16–22]. Accumulating evidence suggests that osteogenic changes in subchondral bone develop before articular cartilage degeneration and that vascularization from subchondral bone to articular cartilage plays a significant role in cartilage erosion in OA [13]. To date, in humans, the effects of exercise on the interplay of articular cartilage and subchondral bone have not been studied. Furthermore, emerging evidence suggests that physiological joint loading could be used to counteract the inflammatory pathways and restore anabolic activities [23]. Biological pathways within a joint are mechanosensitive, and biomechanical factors, such as exercise loading, are modifiable and represent a potential means of intervention.

Quantitative magnetic resonance imaging (qMRI) and cone beam computed tomography (CBCT) allow detailed investigation of articular cartilage and subchondral bone and the effects of moderate-intensity exercise on them [24–31]. In this randomized controlled trial, we plan to investigate the effects of an 8-month joint loading exercise regimen on knee joint subchondral bone morphology and properties, articular cartilage morphology and biochemical alterations, and their 12-month maintenance in women with mild KOA. Furthermore, we will examine the effects of exercise regimen on molecular biomarkers related to OA and inflammation, bone traits, physical function, performance, body composition, and clinically important OA-related symptoms. We will assess changes in these variables after the 8-month intervention, which is the primary timepoint. In addition, we will investigate whether the potential benefits of exercise will be maintained 1 year after the training period. The treatments that will be provided to members of a reference group comply with the current standard rehabilitative management for knee OA patients [32]. The aim of this article is to describe a randomized controlled trial that will investigate the effects of a multicomponent exercise intervention on subchondral bone and articular cartilage in postmenopausal women with mild knee OA.

## Methods and analysis

### Study design

This 20-month study will be an 8-month randomized controlled exercise intervention trial (RCT) with two parallel groups and a subsequent 12-month follow-up after the intervention. After baseline measurements, the participants will be randomly assigned to one of two study arms: a multicomponent exercise group or a reference group. All outcome measures will be performed at baseline, after the 8-month intervention, and at follow-up 12 months after completion of training. The primary outcome measures will be assessed separately, although they assess the same phenomena from different perspectives. The study will be conducted in two separate phases for practical reasons. Recruitment of the participants started in June 2023, and the data collection will be finished by March 2026. All of the data collection will take place at the University of Jyväskylä and Hospital Nova. The protocol described in this study is version 1.0, finalized on October 16, 2024.

### Participants and selection criteria

**Fig. 1:**
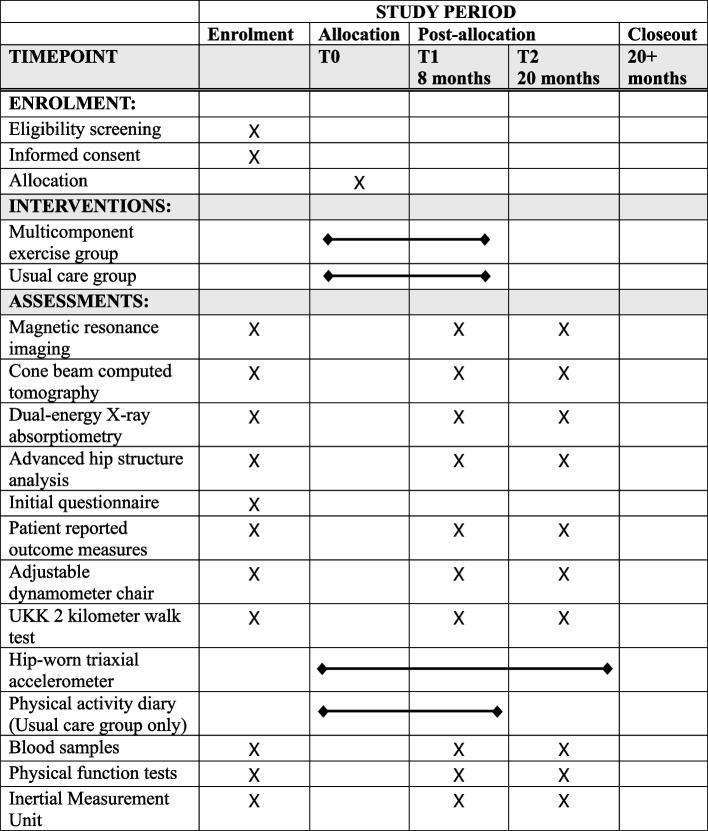
Trial profile

**Fig. 2:**
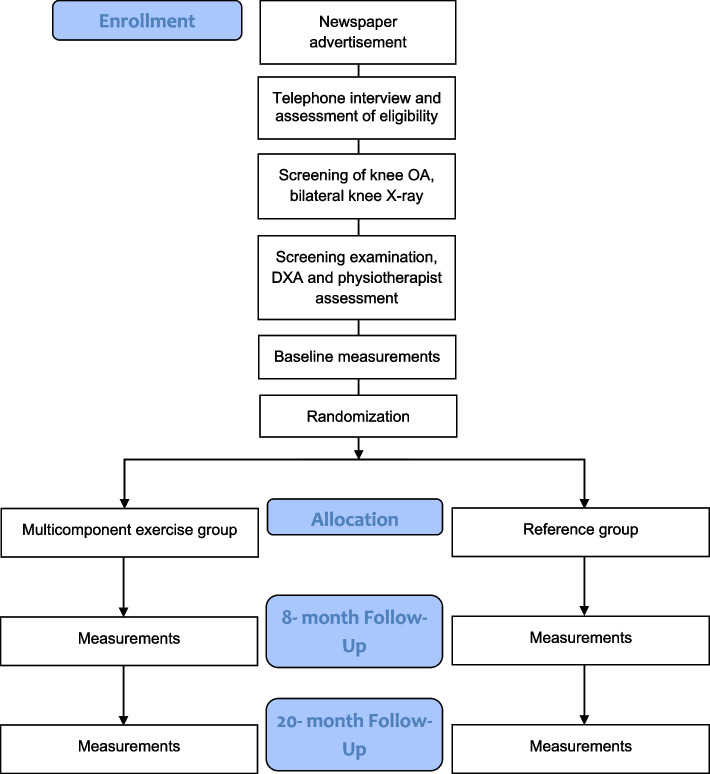
Trial flowchart

Volunteer postmenopausal women between the ages of 55 and 75 will be recruited from the county of Central Finland through local newspaper advertisements. Eligibility will be initially assessed via a structured telephone interview. The telephone questionnaire includes questions about medical history, hormonal replacement therapy status, knee symptoms, and current level of physical activity. Participants who are eligible based on the telephone interview will be invited to weight-bearing radiography of both knees. An experienced fellowship-trained musculoskeletal radiologist will assess the degree of OA in the tibiofemoral joints according to the Kellgren-Lawrence (KL) grading system [33]. Participants with a KL score of I (possible osteophytes) or II (definite osteophytes, possible joint space narrowing) will progress to the next stage of eligibility assessment and undergo physiotherapy screening. Prior to the screening, participants will fill out an initial health questionnaire (Supplement 1). During the screening, any possible physical or medical limitations to full participation in the intervention will be assessed, e.g., possible physical disabilities, excessive laxity of the knee joint, severely restricted joint range of motion, and history of any illness for which exercise is contraindicated or that would limit participation in the exercise regimen. The assessing physiotherapist may refer participants to the study physician if deemed necessary for their safe participation in the study. Participants will be excluded if they meet one or more of the following criteria: body mass index (BMI) > 35 kg/m^2^, knee pain > 7 on the Numeric Rating Scale (NRS, 0–10) in the past month, known loose particles in the knee joint, acute inflammation in knee joint, intra-articular knee steroid injection or oral steroid treatment in the previous 12 months, systemic hormonal replacement therapy, undergoing treatment for osteoporosis or a *T*-score for femoral neck bone mineral density (BMD, g/cm^2^) lower than − 2.5, i.e., indicating osteoporosis as measured by dual-energy X-ray absorptiometry [34], type I diabetes, cardiac disease, kidney deficiency, diagnosed rheumatic disease (other than OA), surgical procedure on the knee (excluding partial meniscectomy or arthroscopy over 12 months ago), or joint replacement surgery in the lower limbs. Additional exclusion criteria are implanted devices that would contraindicate MRI, including electronic or ferromagnetic implants, such as pacemakers; unsuitable metals within the body; artificial aortic heart valves; metal particles in the eyes; or large tattoos on the lower limb. All participants who meet the inclusion criteria will be enrolled in the study and undergo baseline measurements. Trial’s participant selection and measurement procedures are presented in Fig. 1 and Fig. 2.

### Sample size

Sample size and power calculations were estimated for the T2 relaxation time endpoints (s) of this study using Stata (StataCorp, College Station, TX and SPSS, IBM Corporation), as no adequate a priori data exist on subchondral bone or T1ρ estimates in exercise intervention studies. The sample size was evaluated using simulation-based sample size, because no accurate and reliable data was available to make an estimate. The starting values for the simulation were based on data from our previous studies [17, 18]. It is estimated that for 80% statistical power, 38 participants are needed in each group to detect a mean difference of 4 ms in T2 between the groups at significance level of *p* < 0.05. The dropout rate at the 12-month follow-up is estimated to be approximately 15% based on our previous studies [17, 35], so at least 90 participants will need to be recruited.

### Randomization and blinding

Participants will be enrolled by VMK, JM, and research assistants. The enrollment personnel will be blinded to the allocation. After the baseline measurements in each phase, participants will be randomized by an external statistician blinded to the clinical information into one of the two study arms using statistical software and participant ID numbers. A computer-generated block randomization, stratified by KL grade, age, and use of local hormonal replacement therapy, will be used to guarantee an equal distribution of OA severity and other confounders within each group and an equal group size. A random sequence will be generated by a statistician who is not involved in the measurements or recruitment of participants. In an exercise intervention study like this, it is not possible to blind participants to the intervention. Researchers will be blinded to group allocation as well as to the interventions and measurements, with the exception of VMK and JV, who will coordinate the research at an operational level, e.g., managing the scheduling of measurements and acting as contact persons for the participants. Owing to practical limitations, some of the physiotherapists providing the intervention will also perform the physical performance measurements. All of the statistical analyses will be performed by a statistician blinded to the participants and measurements.

### Primary outcomes

#### Magnetic resonance imaging

qMRI contrast mechanisms will be used to assess the tibiofemoral and patellofemoral articular cartilage. Co-primary qMRI outcomes will be T1ρ and T2 relaxation time mapping which will be further enhanced through a texture analysis. qMRI can be used to reveal the architecture of the articular cartilage and indirectly alterations in its mechanical properties [28]. T1ρ and T2, in particular, are sensitive to biochemical changes in cartilage [29–31]. Previous studies have shown that the severity of radiographic knee OA and histological degeneration of cartilage are associated with increased T2 values [31, 36]. In vivo test–retest reliability for T2 measurements, expressed as coefficients of variation (CV), has been reported to vary from 2.3 to 6.5% and from 2.3 to 6.1% for T1ρ [37].

The estimated cartilage glycosaminoglycan content will be measured using T1ρ. T2 relaxation time mapping will be used to provide information about cartilage quality, with longer times reflecting a decrease in collagen matrix integrity and an increase in water content, which are associated with cartilage degeneration [31, 38]. Furthermore, the qMRI measurements will be further enhanced through a texture analysis approach [39] where spatial relationships and texture patterns will be analyzed from tibiofemoral cartilage. Morphometric analysis will be used to analyze articular cartilage thickness and volume.

Prior to imaging, the participant will be advised to refrain from any strenuous physical activity to minimize possible transient changes in the knee cartilage volume and composition [40, 41]. MRI will be performed via 3T MR system (Magnetom Vida, Siemens Healthineers, Erlangen, Germany) at Hospital Nova, and it will include T1ρ and T2 relaxation times followed by clinical series such as pd, t2, t1, and dess. The session will take approximately 45 min. The participants will be imaged while lying supine with the knee stabilized in slight flexion. MRI will be performed using a sagittal multislice multiecho fast spin echo sequence (Table 1). The slices will be positioned perpendicular to a line tangential to the posterior femoral condyles in the axial scout view. Three slices, each covering the central region of the medial and lateral condyles as well as the slice with the thickest cartilage in the transverse plane located in the middle third of the height of the patella, will be analyzed. The knee with the highest degree of OA, as measured by the radiographic KL scale, will be imaged. In cases where both knees have identical KL grades, the more symptomatic knee at the beginning of the study will be imaged.

**Table 1:** MRI parameters

	pd	t2	t1	dess	T2 map	CWt1ρ
Repetition time [ms]	900	1000	400	4.1	1680	3000
Echo time [ms]	76	125	13	5	13.8*n, *n* = 1 to 5	3.18
Flip angle [degrees]	120	120	120	25	180	25
Field-of-view [mm*mm]	180*180	180*180	180*180	150*150	160*160	160*160
Matrix [px*px]	224*224	224*224	224*224	256*256	384*384	256*256
Slice thickness [mm]	0.8	0.8	0.8	0.6	3	3
Spin-lock time [ms]	N/A	N/A	N/A	N/A	N/A	0, 10, 20, 30, 40
Spin-lock frequency [Hz]	N/A	N/A	N/A	N/A	N/A	500

Weight-bearing cartilage regions of interest from single sagittal slices at the center of the medial and lateral tibial and femoral condyles will be segmented via a semiautomated in-house MATLAB application (MathWorks, Inc. Natick, MA, USA). Additionally, cartilage regions of interest from the slice with the thickest cartilage in the transverse plane will be segmented. In case of multiple people performing the segmentation, inter-observer errors will be determined. In our previous study, the inter-observer error expressed as root-mean-square coefficients of variation (CV_RMS_) for the T2 full-thickness region of interest ranged from 1.3 to 3.3% [18].

#### Cone beam computed tomography

CBCT will be used to evaluate subchondral bone mineral density (BMD, g/cm^3^) and content (BMC, g), as well as tibiofemoral joint space narrowing. Subchondral BMD can be measured with approximately 1–2% precision using quantitative CT [24, 25]. In addition, the spatial resolution of CBCT is typically greater than that of a conventional multidetector CT, and the method can be performed while standing to provide information on the joint under loading [26, 27]. The in vivo test–retest reliability CV_RMS_ for weight-bearing CBCTs measuring the femoral subchondral bone thickness has been reported to be 7.1 to 9.4% and for tibial subchondral bone thickness 7.8 to 13.4% [42].

Prior to CBCT, participants will be advised to restrain from any strenuous physical activity to minimize possible transient changes in the subchondral bone and knee cartilage. The CBCT imaging session will take approximately 20 min, with an actual scan time of 16 s. Imaging will be conducted in the natural standing position via a high-resolution twin robotic X-ray system (slice distance 0.4 mm, pixel spacing 0.4 mm × 0.4 mm, energy 116 kV, cylindrical FOV of approx. 25 cm in diameter and height, scan range 0.4 mm × 640, dose area product 55 dGy/cm^2^) (Multitom Rax, Siemens Healthineers AG, Erlangen, Germany) at Hospital Nova. The knee with the highest degree of OA, as measured by the radiographic KL scale, will be imaged. When both knees have identical KL scores, the more symptomatic knee at the beginning of the study will be imaged. All of the primary outcome measures are presented in Table 2.

**Table 2:** Primary outcomes

Measurement	Variables
Magnetic resonance imaging	T1ρ and T2 mapping relaxation times (ms) of tibiofemoral and patellofemoral cartilage. Texture analysis to analyze spatial relationship and texture patterns of tibiofemoral cartilage. Additionally, morphometry analysis to analyze articular cartilage thickness and volume, and a set of clinical series (pd, t2, t1, and dess) will be performed
Cone beam computed tomography	Subchondral bone mineral density (BMD, g/cm^3^) and content (BMC, g), as well as tibiofemoral joint space narrowing

### Secondary outcomes

#### Body composition and femoral neck bone properties

Body composition and femoral neck bone traits will be assessed via dual-energy X-ray absorptiometry (Lunar Prodigy; GE Lunar Healthcare, Madison, WI, USA). Body composition analyses will be carried out using enCORE software (ENcore 2011, version 14.10.022). The total body fat percentage, fat free mass index (FFMI, kg/m^2^), and lean body mass (kg) will be measured using the manufacturer’s software and protocols. The in vivo precision of these measurements has been reported to be CV 0.8–1.3% [43]. The proximal femur areal bone mineral density (aBMD, g/cm^2^) and bone mineral content (BMC, g) will be measured from both sides. The in vivo precision of these measurements has been reported to be CV 1.9% for BMD [44] and 1.4% for BMC [45]. The cross-sectional geometry of the femoral neck will be analyzed using advanced hip structure analysis (AHA) via the manufacturer’s software. These indices will include the cortical thickness (mm), femoral neck width (mm), cross-sectional area (CSA, cm^2^), cross-sectional moment of inertia (CSMI, cm^4^), section modulus (cm^3^), and femoral neck compressive bone strength index [46–48]. The in vivo repeatability of the CSA, CSMI, and section modulus has been reported to vary between CV 2.2 and 6.2% [44, 49].

#### Questionnaires

The participants will be given the option to complete the questionnaires either in printed or electrical form. Electrical questionnaires will be completed via the secure Research Electronic Data Capture (REDCap) web-based software platform designed to support data collection for research studies [50]. Physical questionnaires will be digitized into REDCap by the researchers.

##### Health status

General health will be assessed at baseline via a questionnaire devised by the research group. The questionnaire addresses medical conditions, current medications and supplements, history of menopausal hormone replacement therapy, and previous accidents relevant to the knee joints. During the intervention, participants will be asked to report their daily amount of pain medication taken to manage their knee pain.

##### Self-rated physical activity

Self-rated physical activity will be assessed using the long form of the International Physical Activity Questionnaire (IPAQ) [51]. The questionnaire is a standard tool for gathering globally comparable information on physical activity. There are four domains in the IPAQ series, including household and yard work activities, occupational activity, self-powered transport, leisure-time physical activity, and sedentary activity. The long form of the self-assessed IPAQ has been found to have a test–retest Spearman’s reliability coefficient of 0.82 and criterion validity Spearman’s coefficient of 0.52 when assessed against an accelerometer in the Finnish population [51]. Additionally, among the 12 countries, Spearman’s correlation coefficients for test–retest reliability were observed to be approximately 0.8 [51].

##### Health-related quality of life

The RAND-36-Item short-form health survey instrument [52] will be used to measure self-assessed quality of life. The 36 items of the RAND-36 measure eight different aspects of health, which are physical functioning, role limitations caused by physical health problems, role limitations caused by emotional problems, social functioning, emotional wellbeing, energy/fatigue, pain, and general health perceptions. All items are scored from 0 to 100, and scores within the same scale are averaged to create each scale. Higher scores represent a more favorable health state. The scores represent the percentage of the total possible score achieved. Cronbach’s *α* coefficients describing the internal consistency of the questions in a Finnish sample ranged from 0.80 to 0.94, with the highest being for physical functioning [53].

##### Clinically important OA symptoms and physical function

Self-reported clinically important OA symptoms and physical function will be assessed via the Likert version of the Knee injury and Osteoarthritis Outcome Score (KOOS) [54]. The five dimensions covered in the KOOS are pain, symptoms, activities of daily living, sport and recreation functions, and knee-related quality of life. Each of these dimensions is scored separately and converted to a 0–100 scale representing the percentage of the total possible score achieved. A score of zero represents extreme knee problems, and a score of 100 represents no knee problems. In people with OA, the KOOS exhibits internal consistency for individual subdomains, with Cronbach’s alpha ranging from 0.74 to 0.92, and test–retest reliability, as indicated by the intraclass correlation coefficient (ICC), which is within the range of 0.83 to 0.90 [55]. The reliability of the Finnish version of the KOOS has been found to be comparable to that of its international counterparts [56].

##### Self-rated work disability

The short version of the Örebro Musculoskeletal Pain Screening Questionnaire (ÖMPSQ) [57] will be used to measure self-rated work disability. The ÖMPSQ is a tool developed to assist in the early identification of psychosocial variables and patients at risk of developing work disability due to pain. The short version of the ÖPMSQ has an 85% ability to predict more than 14 days of accumulated sick leave in an occupational sample, and it has been most often used in people with back pain, where it has an internal consistency of 0.80, as measured with Cronbach’s *α* [57]. The questionnaire will only be filled out by participants who are still actively working. In addition, participants will be asked how they would rate their current work ability on a scale from 0 to 10, if their prime work ability was a 10.

#### Physical performance and physical activity measures

##### Muscle strength

The maximal isometric knee extension and flexion strength of both legs will be measured using an adjustable dynamometer chair (Good strength; Metitur Ltd, Jyväskylä, Finland). The measurements will be continued until the results do not increase, with a minimum of 4 repetitions for each direction of movement. The best results will be used and recorded in Newtons. The individual settings of the dynamometer chair will be standardized between the measurements. In our laboratory, the CV of the test has been recorded to be 6.3% for knee extension and 8.5% for knee flexion [58].

#### Aerobic fitness

The maximal aerobic power, VO2 max, will be estimated using the UKK 2-km walk test (UKK Institute, Tampere, Finland) [59]. The test requires participants to walk 2 km at their fastest possible steady pace. VO2 max is estimated by walking time, BMI, age, and heart rate at the end of the test. Heart rate will be measured by a portable heart rate monitor (Polar WearLink W.I.N.D. with Polar RS800CX, Polar Electro Ltd., Kempele, Finland). The test is feasible for estimating VO2 max and has a correlation coefficient of 0.69–0.77 with the maximal effort VO2 max test on a treadmill, indicating reasonable validity [59, 60].

#### Accelerometer measurements

Physical activity, sedentary behavior, number of breaks in sedentary time, standing time, and sleep will be measured in 6-s epochs via a hip-worn triaxial accelerometer (Movesense; Suunto, Vantaa, Finland) with a mean amplitude deviation (MAD) and angle for postural estimation (APE) analysis algorithms (ExSed; UKK Terveyspalvelut, Tampere, Finland) [61–64]. For physical activity, the variables recorded will be the mean daily number of steps and physical activity, which is categorized into light physical activity (1.5–2.9 metabolic equivalent of task (MET)), moderate physical activity (3.0–5.9 MET), and vigorous physical activity (≥ 6.0 METs) on the basis of physical activity intensity. Sedentary behavior will be divided into sitting and lying time, which is recorded as the time spent in these positions with an energy expenditure of less than 1.5 METs. Standing time and breaks in sedentary time will be recorded separately. The time in bed will be recorded using the accelerometer on a wristband and will be divided into three categories (low, medium, and high movement) on the basis of the change in wrist orientation between consecutive epochs when in bed [65].

MAD algorithm has been shown to possess at least 97% sensitivity and specificity in classifying sedentary activities and different intensity levels of walking and running [62]. The combined MAD and APE algorithms have achieved approximately 90% accuracy in classifying body posture into lying, sitting, and standing under free-living conditions [63].

In the intervention group, physical activity, sedentary time, and sleep will be measured throughout the 8-month intervention with the ExSed application (ExSed; UKK Terveyspalvelut Oy, Tampere, Finland), which provides participants with feedback on their physical activity levels, sedentary time, sleep quality, and sleep quantity every time they open the application [64].

The reference group’s accelerometer measurements will be conducted every 2 months for 7-day periods using the Baseline version of the ExSed application (Baseline; UKK Terveyspalvelut Oy, Tampere, Finland), which provides participants with feedback on the number of steps, sleep quality and quantity after the 7-day measurement period without any online information. In addition, after the 8-month intervention, 7-day measurements with the Baseline version will be conducted every 4 months for participants in both groups during the follow-up period. The accelerometers and analysis algorithms are identical between applications.

#### Physical activity diary

During the intervention, the daily physical activity of the reference group will be recorded using a leisure-time physical activity diary. The diary will be completed daily, and each activity, duration, and intensity (1 = light, 2 = moderate, or 3 = vigorous) of physical activity will be recorded. Diaries will be converted into MET-hours per week using a scheme modified from Ainsworth et al. [66].

#### Blood samples

Fasted blood samples will be taken from the antecubital vein via sterile techniques and the samples will be stored at − 80 °C at the University of Jyväskylä Sports Laboratory until further analysis, maximum 5 years. Systemic inflammation markers and the circulating metabolome, including resistin, leptin, adiponectin, lipids, and lipoproteins, will be assessed. Only the laboratory personnel who collect the blood will have access to the pseudonymized samples.

#### Physical function

Physical function will be measured using the Osteoarthritis Research Society International recommended tests: the 30-s chair-stand test, timed up and go test, 40-m fast-paced walk test, and stair climb test, which have been shown to be adequate for measuring changes over time in individuals with knee OA [67, 68]. In addition, static balance will be measured using the single-leg stance test. A supervised practice trial will be conducted to check the safety and understanding of the physical function tests. Two performances of each test will be conducted, and the best result reported. Knee joint pain will be assessed after each test.

The 30-s chair-stand test is conducted with the participant in a seated position with the participant’s feet flat on the floor, shoulder-width apart, and arms crossed over the chest. The participant stands up straight until the hips and knees are fully extended and then sits back down until the buttocks touch the seat completely. Participants are permitted to use their thighs for support if they cannot complete the movement with their arms crossed over their chest. This movement is repeated for 30 s, the number of repetitions is counted, and the need for thigh support is reported. The test–retest ICC of this test has been reported to be 0.89 and it is moderately correlated with leg press performance in community-residing older adults [69]. The inter-rater reliability of the test ranges from 0.93 to 0.98 and the intra-rater reliability ranges from 0.97 to 0.98, as measured by the ICC, in people waiting for hip or knee joint replacement surgery [70].

In the 40-m fast-paced walk test, participants walk as quickly as safely possible, without running, along a 10-m walkway, then turn around a cone, return, and repeat for a total distance of 40 m. The participants turn at a cone placed 2 m beyond each end of the walkway. The total time taken for 40 m is measured via a stopwatch. The inter-rater reliability of the test has been found to be 0.95, as measured by the ICC [71]. Fast-paced walk tests up to 40 m also possess good construct validity and responsiveness in adults with knee OA [72].

In the timed up and go test, participants start from a seated position with their back resting on the back of the armchair, stand up, walk to a mark 3 m away, turn around, and return to sit back in the chair at a regular pace [71, 73]. The total time taken is measured using a stopwatch, and the need for support from the chair is reported. This test has excellent intra-rater reliability with an ICC of 0.97, and an inter-rater reliability ICC of 0.96, and has defined a minimum detectable change of 1.14 s in people with knee OA [74].

In the stair climb test, participants ascend and descend a flight of 16 stairs as quickly as possible but in a safe manner. The height of the steps is 17 cm. Participants are encouraged to use the handrails only if necessary, and their usage is reported. The total time taken is measured with a stopwatch. For people with lower extremity OA, negotiating stairs is a common activity limitation and rehabilitation goal. The stair climb test provides a direct and relatively stable measure of this ability [75].

In the single-leg stance test, participants stand on the leg with lower KL classification, and in the case of matching classifications, they stand on the less symptomatic leg at the beginning of the study. This leg’s heel is raised on the affected leg’s medial shin with the hip rotated outward. The upper extremities hang freely on the sides, and the eyes are kept open. The total time that can be spent in the position is measured with a stopwatch, and the test is stopped at 60 s at the latest. The test–retest ICCs of the single-leg stance tests have been found to be 0.86, and the inter-rater reliability was 0.75–0.85 in older adults [76, 77]. However, the minimum detectable change values for the single-leg-stance-time are rather high at 24.1 s [76].

#### Inertial measurement unit (IMU)

Five IMUs (NGIMU, X-IO Technologies, UK) will be used during the 40-m fast-paced walk test and stair climb test. In addition, the participants will be measured during a 2-min walk at their regular pace. During the 2-min walk, the participant will be asked to stop their movement before the turns to help identify changes in movement within the gathered data. The IMUs will be mounted on the participant’s both ankles, middle of both femurs, and lower back at the level of the umbilicus. The IMUs will be calibrated, and any gaps will be checked in the test data daily before beginning the measurements. The sensors will sample accelerations, gyrations, and magnetic field strengths 100 times per second with measurement ranges of ± 16 multiples of gravitational acceleration, ± 2000°/s, and ± 1300 µT, respectively, via 16-bit analog-to-digital conversion. Data will be recorded over a wireless Wi-Fi connection, and the sensors will be synchronized with each other using the manufacturer’s implementation. The data will be used to analyze the lower body’s segmental acceleration (m/s^2^) and to model flatland and stair walking. All secondary outcome measures are presented in Table 3.

**Table 3:** Secondary outcomes

Measurement	Variables
Dual-energy X-ray absorptiometry	Femoral neck areal bone mineral density (aBMD, g/cm^2^), and bone mineral content (BMC, g), total body fat percentage, fat free mass index (kg/m^2^), and lean body mass (kg).
Advanced hip structure analysis	Femoral neck cortical thickness (mm), width (mm), cross-sectional area (CSA, cm^2^), cross-sectional moment of inertia (CSMI, cm^4^), section modulus (cm^3^), and strength index.
Questionnaires	General health questionnaire at baseline (supplement 1), International Physical Activity Questionnaire, RAND-36-Item short-form healthy survey, Knee injury and Osteoarthritis Outcome Score, short version of the Örebro Musculoskeletal Pain Screening Questionnaire, and rating of current work ability on a scale from 0 to 10.
Dynamometry	Maximal isometric knee extension and flexion muscle strength (Newton).
UKK 2-km walk test	Estimated maximal aerobic power, VO2 max (ml/kg/min).
Triaxial accelerometry	Light, moderate, and vigorous physical activity times; number of steps; standing, lying, and sitting times; number of breaks during sedentary time; total, low, medium, and high movement time in bed.
Physical activity diary	Metabolic equivalent of task (MET)-hours and quantity of painkillers consumed.
Blood samples	Systemic inflammation markers and circulating metabolome, including resistin, leptin, adiponectin, lipids, and lipoproteins.
Physical function tests	30-s chair-stand test (repetitions), timed up and go test (s), 40-m fast-paced walk test (s), stair climb test (s), and single-leg stance test.
Inertial measurement unit	Lower body’s segmental acceleration (m/s^2^), modeling of walking, and stair climb.

Participant retention and loss to follow-up will be improved by reminding participants of upcoming measurements when the accelerometers are distributed to the reference group every 2 months, reminders within the instructed exercise sessions, making every reasonable effort to schedule measurements at compatible times, and if a participant cannot or refuses to participate in some of the measurements, offering them the opportunity to still participate in the other measurements.

### Eight-month exercise intervention

Participants randomized into the intervention group will undergo a multicomponent exercise regimen. They will participate in supervised training three times a week for 8 months. Step aerobics and resistance training programs will alternate fortnightly. Each session will include a 10-min warm-up, a 30-min effective training session, and a 10-min cooldown. The intervention group will be divided into two to form smaller groups for the exercise sessions. Training sessions will be carried out by experienced and trained exercise instructors. Training sessions will be offered six times a week on various days and times to ensure that participants will be able to attend training three times a week. To ensure the intensity and suitability of the regimen, perceived exertion on the BORG 6–20 scale [78] will be assessed after every training session, along with knee pain on the visual analog scale, psychological readiness to train, and possible additional comments.

#### Step aerobics

Step aerobics training will be conducted at the Faculty of Sport and Health Sciences, University of Jyväskylä. The program includes accelerating and decelerating through forward, backward, and sideways movements with stops and turns to music. The choreographies included will use patterns such as basic steps, V-steps, knee lifts, side steps and jumps, and leaps and hops on and off the board. The tempo of the music for all sessions will be set to 120 beats per minute. The difficulty of the movements and the intensity of workouts will be gradually increased by increasing the height of the step boards and increasing the intensity of the movements. The program will be divided into four distinct 8-week phases. The program will start with a progressive orientation phase, which aims to teach participants the techniques of different steps used in the program. Phase 2 will increase the number of hops and the intensity of the steps and increase the board height to 15 cm at week 13. The third phase will increase the number of hops and jumps off the board performed at a height of 15 cm. The fourth phase will introduce a 5-cm increase in the height of the board while decreasing the number of hops. If a participant is unable to progress safely, the board height will be increased individually when it is appropriate and safe to do so. The characteristics of these phases are presented in Table 4.

**Table 4:** Characteristics of step aerobics training in different phases

**Phase**	**Board height**	Number of steps on the board*	**Number of hops**
Orientation (weeks 1–8)	10 cm	100 to 200	0 to 150
Phase 2 (weeks 9–16)	10 cm/15 cm	130 to 250	200
Phase 3 (weeks 17–24)	15 cm	200	180
Phase 4 (weeks 25–32)	20 cm	180 to 190	160

^*^The number of steps shown includes steps on the board only. Various steps that do not include a clear step on the board, i.e., touch steps from the board to the floor and stepping on the board and floor, are not included

#### Resistance training

Resistance training will be conducted at the University of Jyväskylä’s Sport and Health Sciences laboratory, and the program will emphasize training of the following muscle groups: quadriceps, hamstrings, hip abductors, adductors, and extensors. In addition to lower limb exercises, trunk and upper body exercises will be applied at intervals. Resistance training will be conducted mainly using air resistance equipment (HUR Oy, Kokkola, Finland). This equipment will save the participants’ training resistance, and the settings used, as well as increase the resistance automatically. When a participant performs 12 repetitions with a resistance, the system will increase the resistance for the next visit by 2 kg for the leg press and 0.5 kg for the other exercises. The resistance can also be changed manually if necessary. During the first training session, all adjustments for the exercises will be guided, and the starting loads will be determined individually for each participant. The participants will be instructed to aim for 12 repetitions in the exercises using an external load. Progression will follow a 2 + principle. For example, when a participant can perform 2 more repetitions in a set of 3 × 10, more load will be needed. Instructors will be monitoring the progressive increase in resistance during the program. Air resistance exercises will include hip adduction/abduction, trunk flexion/extension, inclined leg press, knee extension/flexion, and push up/pull down. Other exercises will be included depending on the number of participants in the practice. These exercises include hip flexion/extension in machine, bridge, plank, lateral band walk, calf raise, dead bug, squat press, biceps curl, and triceps pushdown. Resistance training will be conducted using cross training and two to three sets of each exercise will be performed. Kinetic exercises will include steady concentric and eccentric phases. A 30- to 60-s rest between the sets will be given. The exercises used in the program are described in more detail in Supplement 2.

### Reference group

The treatments provided to the reference group represent standard rehabilitative management for knee OA patients in Finland. The home exercises focus on functional exercises to maintain lower extremity flexibility and muscle function. The home exercises are described in Supplement 3. The home exercises will be instructed to be carried out three times a week, with each workout lasting 30 min. Members of the reference group will be instructed to keep a diary of their home exercise workouts to assess compliance. Otherwise, they will be asked to maintain their habitual physical activity during the intervention period.

### Twelve-month follow-up period

All participants will be advised to continue spontaneous physical activity for 12 months after the post-intervention measurements, whereas no other specific instructions will be given.

### Assessment of adverse effects

In this study, the expected exercise-related adverse effects may include normal muscle soreness after the strength tests and exercise. Any unanticipated adverse effects of exercise may include various types of sudden muscle and tendon injuries in the lower limbs. The research team includes a specialist medical doctor (JP) who, if necessary, will perform an injury assessment and the need for further treatment. All participants will be covered by the university insurance. The estimated maximum total radiation exposure per patient for the entire study is 0.284 mSv and the actual exposure will be lower. All adverse effects or health issues related to the testing protocol or intervention exercise regimen will be documented and reported in publications using structured MedDRA vocabulary. A visual analog scale (0–100 mm) will be used to measure self-reported knee pain following each individual measurement and training session, along with any other physical symptoms, such as stiffness, pain elsewhere than the knee, and general fatigue.

### Statistical analysis

Data will be analyzed with an intention-to-treat strategy, where all the enrolled participants will be included in the analysis in their randomized groups, regardless of protocol adherence. The mean change from baseline in the outcomes will be compared between the groups. One-way analysis of covariance (ANCOVA) with the 8-month endpoint and 12-month follow-up measurements as dependent variables will be used to assess the intervention effect between the groups. Cartilage and bone traits as outcome variables will be adjusted for baseline values, age, height, and body mass. In the association analyses, correlation coefficients will be calculated via the Pearson or Spearman methods with bootstrap-type confidence intervals. In addition, generalized linear models (GLM) with appropriate distributions and link functions will be provided for each outcome. Compliance with the intervention will be calculated using the following formula: [number of exercise sessions performed]: [expected number of exercise sessions] × 100. Statistical analyses will be performed using up-to-date versions of statistical software (Stata, StataCorp, College Station, TX and SPSS, IBM Corporation). Two-sided *p* values with alpha < 0.05 will be used for all tests, and 95% confidence intervals will be provided when the results are presented. A professional academic statistician blinded to the study groups will conduct the analyses.

### Ethics and dissemination

The Ethics Committee of the Health Care District of Central Finland approved the research protocol on May 23, 2023 (Dnro 1U/2023). Written informed consent will be obtained from all participants before their participation in the study by the researchers. All recruited participants will volunteer and be given enough information and time to think before enrolling in the study. Potential participants will receive information sheets and consent forms before the first visit, where they are able to have an informed discussion with the researchers. All of the enrolled participants have the right to withdraw from the study without needing to provide a reason for withdrawal. All participants are insured through collective insurance and have the possibility to consult a physiotherapist, and an attending physician will be consulted if necessary.

The study will be conducted in accordance with good clinical and scientific guidelines and the Declaration of Helsinki. Major protocol amendments, which may impact trial or participant safety, will be openly communicated to relevant parties, such as the Ethics Committee, researchers, participants, and trial registries. The results of the study will be published in peer-reviewed journals regardless of the magnitude or direction of the effect. Every effort will be made to reduce the amount of time between the completion of data collection and the publication of results. Authorship of manuscripts will be determined by the Committee of Medical Journal Editors’ criteria for authorship. No external professional writers will be used. The funding source had no role in the design of this study and will not have any role during its execution, analyses, interpretation of the data, or decision to submit results.

### Data management

All data will be stored in a pseudonymized form on the University of Jyväskylä’s highly secure network folder, which has been specifically tailored for handling sensitive data. Each participant will be assigned a unique ID number, which will be used in all study records and samples. Documents such as logbooks, forms, and any other listings that connect participant ID numbers to other personal data will be kept in a locked file in a location with limited access. The principal investigator will have access to the datasets, and the pseudonymized data will be shared within the group as necessary. A data monitoring committee is not needed, as there are no ethical reasons to stop the trial early. Individual participant data cannot be anonymized and will thus remain restricted to ensure personal data protection. The basic trial-level discovery metadata of the datasets will be made openly available in the University of Jyväskylä’s repository with a DOI for permanent findability and accessibility. Research data will be anonymized by the end of 2030. Methods such as consistency checks against data already stored in the database and range checks will be used to ensure accurate and complete data.

### Patient and public involvement

Patients and the public were not involved in the development of this research protocol.

### Trial status

The recruitment of trial participants has been completed. The second phase of the intervention started in April 2024 and will continue until December 2024. The 8-month post-intervention measurements of phase 1 were conducted, and the participants will be invited for 12-month follow-up measurements in April 2025. Twelve-month follow-up measurements for the second phase will be conducted in December 2025.

## Discussion

This paper describes randomized controlled trial investigating the effects of a progressive multicomponent exercise regimen on subchondral bone, tibiofemoral and patellofemoral cartilage, circulatory biomarkers, femoral neck bone traits, body composition, and physical function in postmenopausal women with mild knee OA.

Knee joint loading exercise does not appear to be harmful to cartilage in people at increased risk of or with knee OA [20]. In OA, there are well-described progressive destructive changes in the articular cartilage, which parallel changes in the underlying bone [79]. Considering the crosstalk between bone and cartilage as a factor in OA initiation raises the question of whether treatments for OA could be directed at modifying this crosstalk by inhibiting disease-related changes in these tissues.

Previous studies have shown that a combination of resistance training and step aerobic exercise prevents functional decline and bone fragility in home-dwelling elderly women [80]. In patients with knee OA, different types of aerobic exercise combined with resistance training appear to have beneficial effects on pain and function [81]. Additionally, the combination of aerobic and strength training did not result in significant changes in self-reported physical function, pain, or joint space width but did result in significant improvements in 6-min walk distance compared with the lifestyle education group, who received attention, social interaction, and health education [82]. Notably, only the first 4 months of the 18-month intervention were facility-based, and strength and aerobic training were delivered within the same exercise sessions. These findings partially support the hypothesis that it is possible to maintain good physical function through a multicomponent exercise regimen and thus postpone age-related functional problems.

To our knowledge, there have been no publications investigating the effects of exercise on the interplay of subchondral bone and articular cartilage in people with knee OA. The aim of this study is to investigate the effects of a multicomponent exercise regimen on the subchondral bone and articular cartilage of the knee joint and the potential interactions between these tissues. With the information gained, we will be better able to prescribe multicomponent exercise regimens for the treatment of mild knee OA and better understand the effects of exercise on subchondral bone and the biochemical properties of cartilage.

## Supplementary Information

Supplementary Material 1.

Supplementary Material 2.

Supplementary Material 3.

Supplementary Material 4.
