# Supplementary material for: Effects of a multicomponent exercise regimen on subchondral bone and cartilage in postmenopausal women with knee osteoarthritis: protocol for a randomized controlled trial
Source: Trials. 2025 Jun 23;26:222. doi: 10.1186/s13063-025-08928-1 (PMC12186390; doi:10.1186/s13063-025-08928-1)
Supplement: Supplementary file 1 — Supplementary Material 1. [file 13063_2025_8928_MOESM1_ESM.pdf]

Name: ____ Date: ______________________

Initial questionnaire

Instructions

Please answer all questions carefully. Depending on the answer options, circle or check the most appropriate option and/or write the answer in the space provided. For questions where there are several options, circle **only one** item in each horizontal row that you consider the most appropriate. If you need to correct any of your answers, please tick over the incorrect entry.

Background information

1. Age _________ years

2. Height _________ cm

3. Weight _________ kg

4. Education

1. Elementary school
2. Primary/Middle School
3. High school
4. vocational school
5. Vocational college
6. Higher education

5. How many years have you attended schools in total? _________ years

6. Are you currently?

1. Retired
2. Unemployed
3. In working life,
   occupational title____
   Main work assignment____

7. How long have you been working? _________year

NUTRIENTS AND STIMULANTS

8. In the past year, have you used:

a) calcium tablets
1. No
2. Yes, which product? ____
   How much? ____

b) vitamin products
1. No
2. Yes, which? ____
   How much? ____

c) natural products
1. No
2. Yes, which? ____
   How much? ____

9. Do you currently have a special diet?
1. No
2. Yes, which?
   a) vegetarianism
   b) lactose-free
   c) gluten-free
   d) something else, please specify ______________________________

10. How many cups of coffee or tea do you usually drink a day?

coffee: ______ cups
tea: ______ cups

11. Have you ever smoked regularly (at least 1 cigarette / day)?

1. I have never smoked regularly
2. I quit regular smoking ______ years ago
3. I have smoked regularly for a total of ______ years, ______ cigarettes / day

12. How often do you drink beer, wine or other alcoholic beverages? Try to include also those times when you only consume small amounts, e.g. a bottle of medium beer or a splash of wine.
- ☐ never
- ☐ about once a month or less
- ☐ 2-4 times a month
- ☐ 2-3 times a week
- ☐ 4 times a week or more

13. How many servings of alcohol have you usually had on the days you consumed alcohol?
- ☐ 1–2 servings
- ☐ 3-4 servings
- ☐ 5-6 servings
- ☐ 7–9 servings
- ☐ 10 or more

14. How often have you drunk six or more servings at a time?

- ☐ Never
- ☐ less than once a month
- ☐ once a month
- ☐ once a week
- ☐ daily or almost daily

15. Symptoms

Please check the box if you have experienced any of the following symptoms:

- chest pain or tightness in the chest ☐ No ☐ Yes
- pain or discomfort in the upper abdomen, chest, neck, shoulders or upper limbs at rest or with increased exertion ☐ No ☐ Yes
- feeling of lack of oxygen or abnormal shortness of breath ☐ No ☐ Yes
- dizziness or disturbance of consciousness during exercise ☐ No ☐ Yes
- symptoms of cardiac arrhythmias (uncomfortably strong, fast or irregular rhythm, bumps) at rest or with increased exertion ☐ No ☐ Yes
- asthenia and fatigue during or after exercise ☐ No ☐ Yes
- swelling in the joint or limb, pain in the limb at rest or increasing with exertion ☐ No ☐ Yes
- severe or prolonged spinal pain radiating to the lower or upper extremities, increasing limb weakness during exertion ☐ No ☐ Yes

16. Illnesses and injuries

Please check the box to see if you have had any of the following:

- history of myocardial infarction ☐ No ☐ Yes
- angioplasty, bypass surgery or other heart surgery ☐ No ☐ Yes
- pacemaker, arrhythmia, heart valve disease, or myocardial disease ☐ No ☐ Yes
- heart failure ☐ No ☐ Yes
- stroke ☐ No ☐ Yes
- type I diabetes ☐ No ☐ Yes
- type II diabetes ☐ No ☐ Yes
- circulatory disease of the lower extremities ☐ No ☐ Yes
- kidney disease ☐ No ☐ Yes
- epilepsy, multiple sclerosis, Parkinson's ☐ No ☐ Yes
- rheumatoid arthritis or ankylosing spondylitis ☐ No ☐ Yes
- osteoporosis or bone fracture risk caused by another disease (e.g. cancer) ☐ No ☐ Yes
- slipped disc radiating to the lower or upper limb ☐ No ☐ Yes
- stress fracture or recent accident ☐ No ☐ Yes
- asthma, COPD or other respiratory diseases ☐ No ☐ Yes

Gynecological anamnesis

17. How many births have you had? ________births.

18. The first menstruation began ________year old.

19. The last menstruation (menopause) was ________year old.

20. Have your ovaries been removed by surgery? ☐ Yes ☐ No

⇨ if yes, why ______________________
⇨ If yes, at what age __________________________

21. Has your uterus been removed by surgery? ☐ Yes ☐ No

⇨ if yes, why ______________________
⇨ If yes, at what age _____________________

Hormonal contraception (timing as accurate as possible)

22. Have you ever used hormonal contraception?

1. birth control pills	No	Yes, from year _______ to year________
2. birth control patches	No	Yes, from year _______ to year ________
3. vaginal ring	No	Yes, from year _______ to year ________
4. intrauterine device	No	Yes, from year _______ to year ________

23. Have you ever used progestin?

1. Lugesteron®	No	Yes, from year _______ to year________
2. Provera®	No	Yes, from year _______ to year________
3. Depo-Provera®	No	Yes, from year _______ to year________
4. Farlutal®	No	Yes, from year _______ to year________
5. Gestapura ®	No	Yes, from year _______ to year________
6. Orgametril®	No	Yes, from year _______ to year________
7. Primolut-N®	No	Yes, from year _______ to year________
8. Primolut-Nor ®	No	Yes, from year _______ to year________
9. Terolut ®	No	Yes, from year _______ to year________
10. Mirena IUD®	No	Yes, from year _______ to year________

11. Something else, what? ___________________, from year _______ to year________

Use of hormone replacement therapy (timing as precise as possible)

24. Have you ever used hormone replacement therapy (tablets, gel, patches) after menopause or ovarian removal surgery?

1. I have never used hormone replacement therapy
2. I have previously used hormone replacement therapy, from year ____ to year ____
3. I still use hormone replacement therapy, since year _____.

25. If you are using or have used hormone replacement therapy, which product have you used and when?

1. Tablet taken orally
   Name of the product ___________________, from year ______ to year _______.
2. Gel product applied to the skin
   Name of the product ____________________, from year ______ to year _______.
3. Hormone patch
   Name of the product _____________________, from year ______ to year _______.

Knee anamnesis (These questions are used to assess the more symptomatic side being examined.)

26. Have you had any injuries to your knee in the past and have you received surgical treatment for them

1. There have been no accidents
2. There have been accidents, but I have not received surgical treatment for them
3. There have been accidents and I have received surgical treatment for them

27. Has doctor previously determined that you have a knee area injury

1. No
2. Yes, which of the following?
   a) Meniscus injury
   b) Ligament injury
   c) Patellar dislocation
   d) Something else, what: ____

28. Have you been diagnosed with osteoarthritis of any joint?

1. No
2. Yes, which joint? ________________________
   When was it diagnosed? _____________________________

29. Have you had any symptoms in the knee area during the past week?

1. No
2. Yes, which of the following?
   a) pain*
   b) stiffness when moving the joint
   c) crackling sound during movement
   d) decreased joint mobility
   e) muscle weakness in the thigh area
   f) swelling in the joint area
   g) other symptoms, what? _______________________

*If you have experienced pain in the knee area during the past week, please check the box that best describes the average **intensity** of the pain over the past week**.**

☐	☐	☐	☐	☐	☐	☐	☐	☐	☐	☐
0	1	2	3	4	5	6	7	8	9	10
No pain										Worst pain imaginable

*If you have experienced pain in the knee area during the past week, please check the box that best describes **how disturbing** you have experienced the pain on average over the past week.

☐	☐	☐	☐	☐	☐	☐	☐	☐	☐	☐
0	1	2	3	4	5	6	7	8	9	10
Does not disturb										Disturbs a lot

30. Do you think knee pain limits your ability to function in any way?
1. No
2. Yes, how? ____

31. Have you received any treatments for the knee area in the last 12 months?
1. No
2. Yes, which of the following:

a) cold packs _____ times
b) acupuncture _____ times
c) joint treatment _____ times
d) exercise _____ times
e) electrotherapy _____ times
f) ultrasound _____ times
g) massage _____ times
h) other treatment, what? ____

32. Do you consider that the treatments received have been beneficial?
1. No
2. Yes, what benefits? ____

33. Do you treat your knee disease in any way?
1. No
2. Yes, in what way? ____

____

34. Have you ever taken medication to treat osteoarthritis of the knee or knee pain?

1. No
2. Yes, what?
   a) Paracetamol, such as Panadol® or Paracetamol®
   b) NSAIDs such as Ibuprofen® or Aspirin®
   c) opioids, such as Tramadol® or Panacod®;
   d) topical NSAIDs that are absorbed through the skin, such as Felden® or Mobilat® gel
   e) something else, what? ____
   When was the last time you took medication? _________________________
   How long did you take the medication? ______________ weeks/months/years

35. Have you been given any medicine by injection into your knee joint during the last 12 months?

1 No
2. Yes, what medicine?
   a) Hyalgan®
   b) Artzal®
   c) I don't know
   How long has it been since the last injection? _____ months ____ weeks

36. Have you taken oral glucosamine medication for knee pain?

1 No
2. Yes, what?
   a) Arthryl®
   b) G-Lenk®
   c) Glucadol®
   d) Glucosamin®
   e) Movere®
   f) something else, what? ____
   When was the last time you took medication? _________________________
   How long did you take the medication? ______________ weeks/months/years

37. Have you ever had an endoscopy of your knee joint?

1. No
2. Yes, when? __________________________
   Was there a procedure performed during the endoscopy?
   a) No
   b) Yes, which one? _______________________
   c) I don't know

38. Have you had more than one computed tomography or X-ray examination in the last five years?

1. No
2. Yes, what imaging and how many times? ______________________________

____

Detailed information required for the health examination

Chronic diseases

39. Have you been diagnosed with musculoskeletal disorders? ☐ no ☐ yes

- What and when

____
____
____

40. Have you been diagnosed with fractures during your lifetime? ☐ no ☐ yes

Shin fracture ☐ no ☐ yes, when______, ☐ Left ☐ Right

Thigh fracture ☐ no ☐ yes, when______, ☐ Left ☐ Right

- other fracture, which and when

____
____

41. Have you been diagnosed with breast cancer? ☐ no ☐ yes

when and how was it treated

____
____
____

42. Have you been diagnosed with any other disease? ☐ no ☐ yes

What and when

____
____
____

43. Do you take NSAIDs or other painkillers? no ☐ yes ☐

**Name of the medicine:**	**Strength**	**Dose**
E.g.		
Burana	400 mg	1 tab in the morning and evening if necessary
__________	________	________________
__________	________	________________
__________	________	________________

44. Do you currently have medication to treat osteoporosis or bones?

1. No
2. Yes, what?
   a) Miacalcic®
   b) Fosamax®
   c) Fosavance®
   d) Didronate®
   e) Alendronat®
   f) Bonviva®
   g) Actonate
   h) Actonate Septitum
   i) Protelos
   g) Bonefos
   h) Evista
   k) something else, what? ______________________________

How long have you been taking the medication? _________ weeks/months/years

45. Do you take cortisone medication in tablet form? no ☐ yes ☐

**Name of the medicine:**	**Strength**	**Dose**
E.g.		
Medrol	5 mg	1 tabl in the morning
____________	__________	______________________________
____________	__________	______________________________

List the other medicines you are using here:

**Name of the medicine:**	**Strength**	**Dose**
E.g.		
Metformin	500 mg	2 tabl in the morning and afternoon
___________	_________	______________________________
___________	_________	______________________________
___________	_________	______________________________
___________	_________	______________________________
___________	_________	______________________________

46. Does your health status limit or prevent you from participating in the study?

– musculoskeletal disease or symptoms:
- ☐ limits or prevents
- ☐ does not limit

– heart and circulatory disease or symptoms:
- ☐ limits or prevents
- ☐ does not limit

– chronic lung disease
- ☐ limits or prevents
- ☐ does not limit

– other disease (e.g. Parkinson's disease, multiple sclerosis, unilateral paralysis) or symptoms:
what ____

- ☐ limits or prevents
- ☐ does not limit

47. If you are willing to participate in the study, how do you assess your chances of participation in the study?

- ☐ My chances are good
- ☐ It is expected that home, work, travel, etc. obligations limit my participation.

THANK YOU FOR YOUR ANSWERS!
