# Supplementary material for: Effects of a multicomponent exercise regimen on subchondral bone and cartilage in postmenopausal women with knee osteoarthritis: protocol for a randomized controlled trial
Source: Trials. 2025 Jun 23;26:222. doi: 10.1186/s13063-025-08928-1 (PMC12186390; doi:10.1186/s13063-025-08928-1)
Supplement: Supplementary file 2 — Supplementary Material 2. [file 13063_2025_8928_MOESM2_ESM.pdf]

Supplementary 2. Intervention group’s strength exercises.

Hip adduction/abduction	To perform hip adduction, participant sits on a machine with legs spread apart and brings them together. To perform hip abduction, participant sits on a machine with their legs together and pushes them apart against the machine’s air resistance.	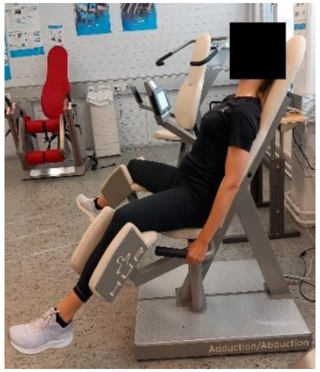	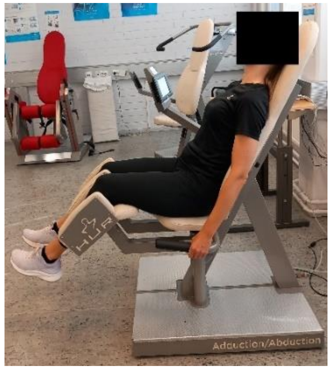
Trunk flexion/extension	Participant sits on a machine and performs trunk flexion and extension against the machine’s air resistance.	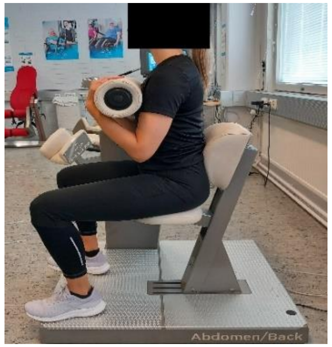	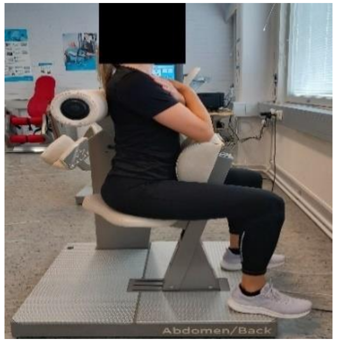
Incline leg press	Participant sits on a machine with back against a pad and feet on a platform. Participant pushes themselves away from the platform with their legs against the machine’s air resistance.	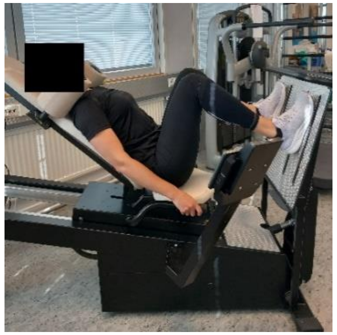	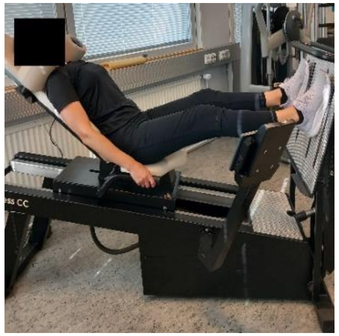

Knee extension/flexion	Participant sits on a machine with legs hanging off the edge and extends or flexes their knees against the machine’s air resistance.	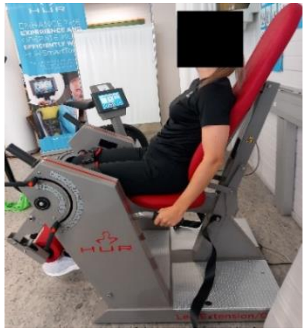	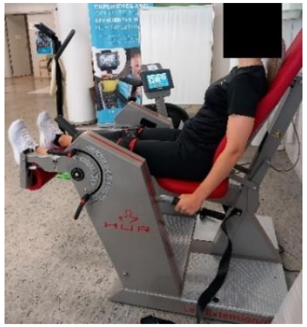
Push up/pull down	To perform push up, participant sits on a machine with handles next to their shoulders, then extends their arms. To perform pull down, participant sits on a machine with handles above their head and pulls the handles down towards their chest.	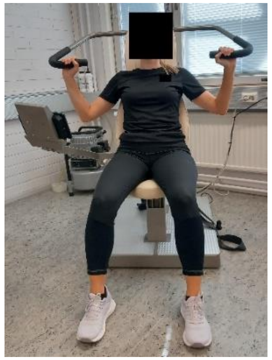	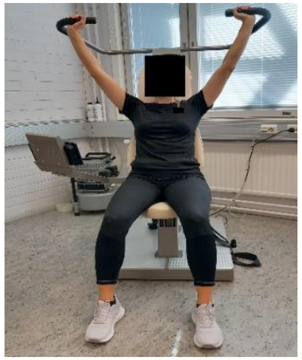

Hip flexion/extension	To perform hip flexion, participant stands on a machine with their working leg hanging off the edge and lifts the leg towards their chest against the machine’s resistance. To perform hip extension, participant stands on a machine with their leg hanging off the edge and pushes their leg backwards against the machine’s resistance.	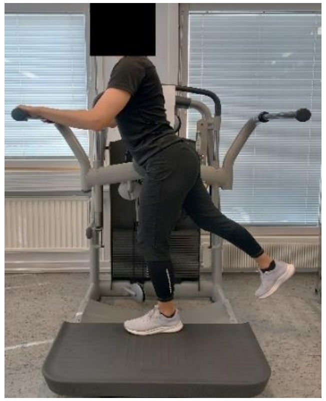 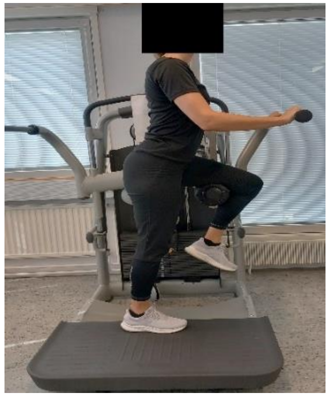
Bridge	Participant lies supine with their knees bent and their feet flat on the ground. Participant lifts their hip off the ground until the body forms a straight line from the shoulders to the knees.	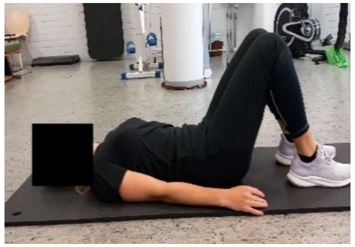 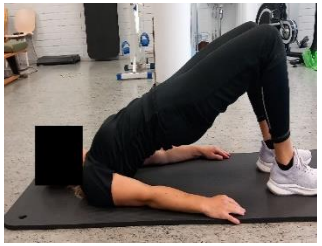

Plank	Participant holds their body in a straight line from head to heels while on their forearms and toes.	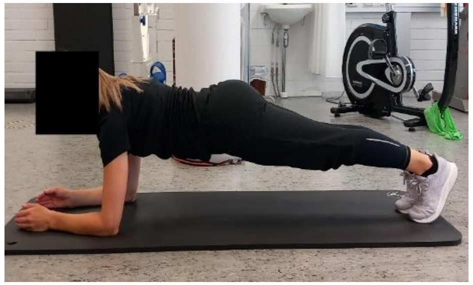
Calf raise	Participant stands on a platform with their heels hanging off the edge, raises their heels as high as possible, and then lowers them back down.	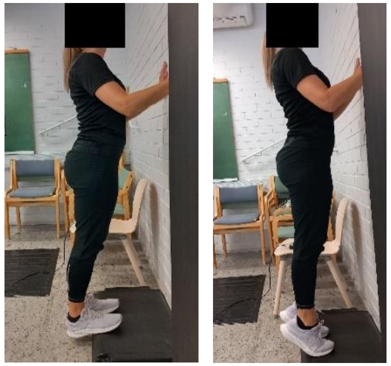
Dead bug	Participant lies supine with their arms and legs in the air. Participant lowers their opposite arm and leg towards the ground and then returns to the starting position.	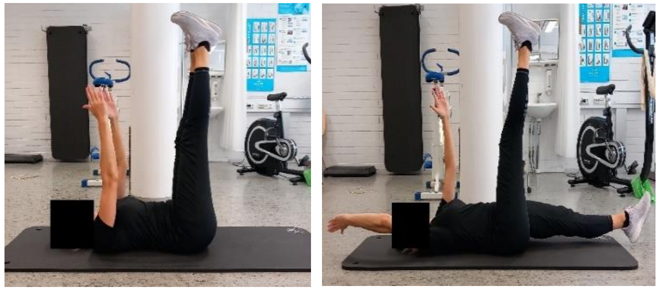

Squat press	Participant squats down as low as possible with dumbbells, stands back up, and lifts the dumbbells or kettlebell above their head.	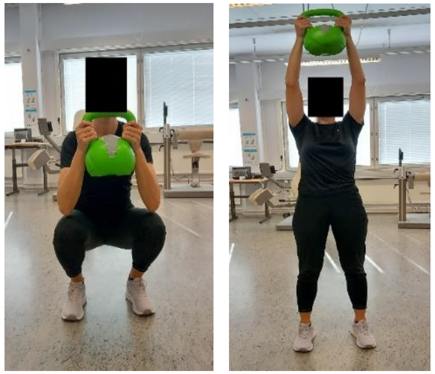
Bicep curl	Participant stands with dumbbells in each hand and curls the weights towards their shoulders.	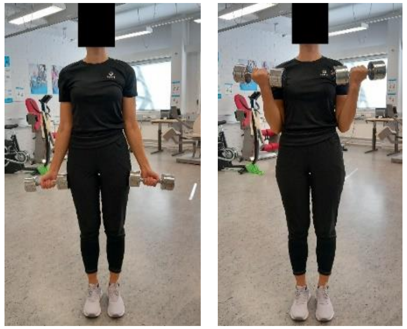
Triceps pushdown	Participant pushes the handle down towards their thighs in a cable machine against resistance.	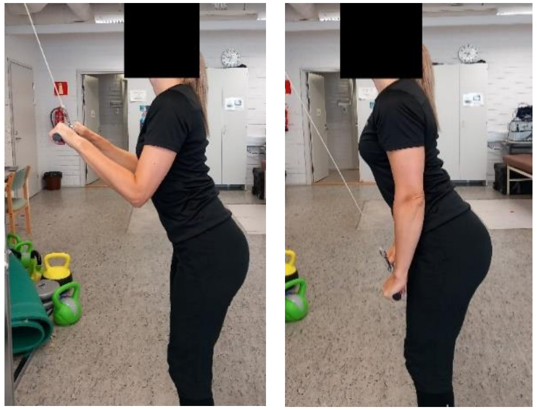
