# Supplementary material for: Effects of a multicomponent exercise regimen on subchondral bone and cartilage in postmenopausal women with knee osteoarthritis: protocol for a randomized controlled trial
Source: Trials. 2025 Jun 23;26:222. doi: 10.1186/s13063-025-08928-1 (PMC12186390; doi:10.1186/s13063-025-08928-1)
Supplement: Supplementary file 3 — Supplementary Material 3. [file 13063_2025_8928_MOESM3_ESM.pdf]

Supplementary 3. Reference group’s home exercises

| | | |
|---|---|---|
| Bench supported squat | Participant takes support from the chair with both hands and slowly squats down while pushing the hips back and keeping the knees behind the toes. No rotation of the knees is allowed during the exercise.<br><br>3 sets of 10 repetitions are performed. | 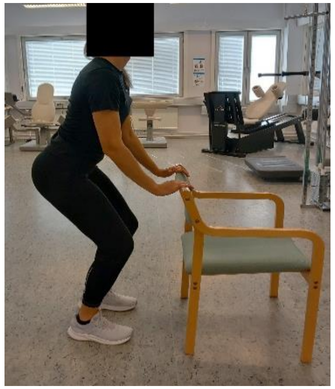 |
| Lying knee extension with pillow under the knee | Participant lies supine with the non-working leg flexed. A pillow is placed under the working leg and the knee is extended while the ankle is dorsiflexed. The contraction is held for 5 seconds before the leg is slowly lowered. 2 to 5 kg of ankle weight can be used.<br><br>3 sets of 10 repetitions are performed. | 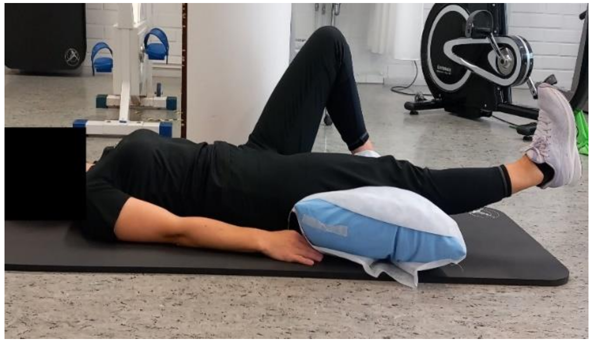 |
| Bridge | Participant lies supine with their knees bent and their feet flat on the floor. Participant lifts their hips off the floor until the body is in a straight line from the shoulders to the knees.<br><br>3 sets of 10 repetitions are performed. | 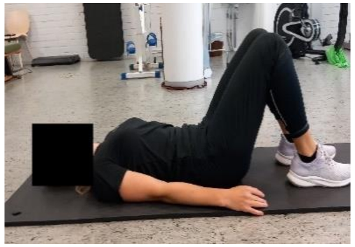 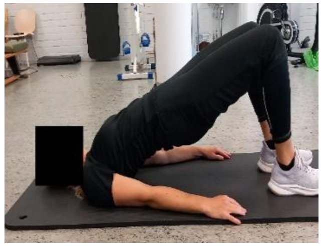 |

| | | |
|---|---|---|
| Dynamic supine hamstring stretch | Participant lies supine with non-working leg straight on the mat. Working leg is flexed from the hip to a 90-degree angle. Knee is extended and relaxed again with a grip behind the knee.<br><br>3 sets of 10 repetitions are performed. | 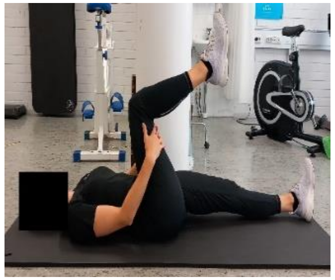 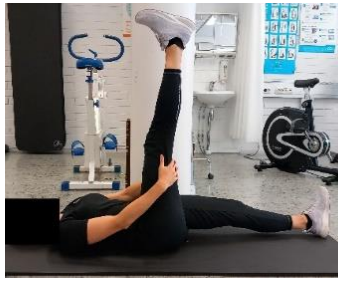 |
| Hip flexor and knee extensor stretch | Participant stands and takes support from the chair while flexing the knee with grip from the ankle. Heel is pulled towards the buttocks so that the knee points straight towards the floor. Stretch is held for 30 seconds.<br><br>2 sets of 3 repetition are performed. | 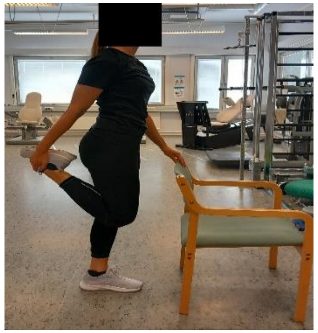 |
